# Correlation of Before and After Invasive Breast Cancer Neoadjuvant Chemotherapy for NFkB, Cyclin D1, and Survivin Expression

**DOI:** 10.30699/ijp.2023.562935.2983

**Published:** 2023-06-20

**Authors:** Primariadewi Rustamadji, Elvan Wiyarta, Ineke Anggreani

**Affiliations:** 1 *Department of Anatomic Pathology, Faculty of Medicine Universitas Indonesia-Dr. Cipto Mangunkusumo National Hospital, Jakarta, Indonesia*; 2 *Department of Medical Sciences, Faculty of Medicine Universitas Indonesia-Dr. Cipto Mangunkusumo National Hospital, Jakarta, Indonesia*

**Keywords:** Breast cancer, Chemotherapy, Cyclin D1, Invasive, NFkB, Prognosis, Survivin

## Abstract

**Background & Objective:**

Patients undergoing neoadjuvant chemotherapy (NC) for invasive breast cancer (IBC) therapy need biomarkers to track their progress. Because of the relationship between NFkB, Survivin, and Cyclin D1 with NC resistance, the different expression levels of each of these biomarkers can be different between pre- and post-NC in IBC. However, no research has examined the correlation between these biomarkers before and after the NC expression. This study aimed to determine the correlation among them.

**Methods:**

Biomarkers expression (low and high) was used to classify 30 samples. ER, PR, HER2, Ki-67 status, tumor grade, age, and NC response were assessed. The amounts of Survivin, Cyclin D1, and NFkB were evaluated using immunohistochemistry, and the samples were classified based on the cut-off. Chi-square and linear regression were used to evaluate the data.

**Results:**

No significant association was found with the changes in the expression of Survivin, Cyclin D1, and NFkB, both before and after the NC. Significant moderate correlations were shown between before and after the NC Survivin expression (r = 0.513) and Cyclin D1 expression (r = 0.543). The correlation between expression of NFkB before and after the NC was not significant.

**Conclusion:**

The high potential of these proteins as prognostic indicators was demonstrated by the strong positive association between the expression of Survivin and Cyclin D1 before and after the NC. This upregulation of biomarkers indicates chemoresistance in developing IBC in the presence of NC.

## Introduction

Among all female malignancies, breast cancer is the most lethal cancer. It accounts for the 11.7% of all diagnoses and 6.9% of fatalities in the United States (1). Malignant tumors of the breast can be either invasive or non-invasive. Among all types of breast cancers, invasive breast carcinoma (IBC) is the most abundant (2-4). Chemotherapy is a crucial aspect of the modern IBC treatment, especially neoadjuvant chemotherapy (NC), provided before surgery (5). NC is the standard care for the patients with locally spreading breast cancer, and it is also the most popular option for the patients with early-stage disease that can be surgically removed (5). 

Cancer cells can defend themselves against damaging effects of NC by activating certain metabolic pathways. The regulatory involvement of NFkB in several anti-apoptotic genes facilitates NFkB ability to produce chemotherapy resistance (6). Some genes promote cell life, such as Cyclin D1; some prevent cell death, such as Survivin; and some promote cell survival, such as x-IAP (6-8). This mechanism significantly contributes to the survivin ability to prevent cell apoptosis (9). Cyclin D1 has a crucial function as a pro-survival gene in triggering resistance mechanisms (6-8). The strong association between NFkB, Survivin, and Cyclin D1 suggests an interrelated role for the NC resistance.

Due to of the association of NFkB, Survivin, and Cyclin D1 with NC resistance, the expression level of each of these biomarkers can be different at pre- and post-NC in IBC (10). Analysis of expression levels before and after the NC is critical because these findings may support the application of these biomarkers as predictive markers of NC response in IBC (11, 12). However, no research has examined the correlation between these biomarkers expression before and after the NC so far. This study aimed to determine the correlation among these biomarkers. We hypothesized that pre-NC levels of these biomarkers are correlated with their post-NC levels. To use these biomarkers as prognostic indicators for the NC response in IBC, this result is anticipated to serve as the foundation.

## Material and Methods

**Design of Study**

This observational cohort research was conducted at the Laboratory of Pathological Anatomy Facility in Universitas Indonesia, from January to June 2022. The Medicine Ethics Committee at Universitas Indonesia approved the methods outlined in protocol number 21111252 in November 2021. Every patient who participated in the study consented to it in writing after being informed of its purpose—methods used in the study ethically following the World Medical Association Declaration of Helsinki (13). This five-year study period began in January 2019 and ended in June 2021, with all data coming from internal administrative files between January 2014 and June 2016. Information on the patient tumor size, tumor grade, age, axillary lymph node metastasis (ALNM), Ki-67 status, HER2 status, lymphovascular invasion (LVI), NC management state (before or after), and NC reaction (partial or complete) was collected. Quantitative findings from the IHC labeling of the paraffin block were also gathered for the information on the expression of Survivin, Cyclin D1, and NFkB.

**Samples**

Paraffin blocks of the primary tumors were acquired from the patients who underwent breast surgery and were initially identified with IBC via histopathology. The specimens from individuals with non-IBC diseases, systemic illnesses (such as hypertension and diabetes), and broken paraffin blocks were excluded from the study. The samples were divided based on the NC management state (before or after). The selected dataset represents the largest representative selection possible from the available departmental records. Only one researcher (EW) had access to the final groupings to avoid any potential prejudice. Other researchers did not have access to the results until the end of the analysis.

**Preparation of Slides**

The coloring method was adapted from Kusmardi et al. (2021) and Primariadewi et al. (2021) (4, 14-16). In brief, the tissue was deparaffinized in xylol (Merck, Jakarta, Indonesia) and rehydrated for 5 min, per the standard operating procedure. Tris-EDTA (Merk, Jakarta, Indonesia) was used at 96°C Decloaking Chamber for 20 min to heat-induce antigen recovery at pH 9.0. The slides were rinsed in phosphate-buffered saline (PBS) at pH 7.4 for 15 min after antigen extraction and then treated with peroxidase for 15 min. The plate was treated with anti-Survivin, anti-Cyclin D1, and anti-NFkB antibodies for 1 hr, then with post-primary and Novolink polymer antibodies for 1 more hr. Before being examined under microscope, tissue sections were stained with a dark chromogen called diaminobenzidine (Abcam, Jakarta, Indonesia), then counterstained with hematoxylin (Merk, Jakarta, Indonesia), followed by 5% lithium carbonate (Abcam, Jakarta, Indonesia).

**Quantification of Survivin, Cyclin D1, and NFkB Expression**

Two experts in pathology, PR and IA, assessed the staining processes. Each sample was inspected under 400x total magnification using a Leica DM750 microscope outfitted with Leica LAZ EZ software and then shot using a computer running the software. Five hundred tumor cells were chosen from five different vision areas, and their expression of Survivin, Cyclin D1, and NFkB was quantified. Each location had at least one hundred malignant cells. Tumor cells were stained brown to reveal membrane and cytoplasmic expression of Survivin, Cyclin D1, and NFkB (17-19). Each field of view was analyzed using cell counter, and the results were categorized as no staining, low positive, positive, or highly positive (20). Quantifying the expression of Survivin, Cyclin D1, and NFkB was done with the H-score (21). The combined efforts of two researchers (PR and IA) determined H-scores for the entire group. To avoid bias, the r calculations were sent to another researcher (EW) who continued working on the sample until thorough examination. The combined H-score from the two researchers were used for the next analysis. 

**Statistical Analysis**

Data were arranged into a master spreadsheet in Microsoft Excel (Microsoft Corp, Redmond, US). The collected data was analyzed in the Statistical Package for the Social Sciences version 20 (IBM Corp, Armonk, NY, USA). The expression levels of Survivin, Cyclin D1, and NFkB were all classified using the median H-score (3). The total H-score from the two researchers was used to determine the expression level of Survivin, Cyclin D1, and NFkB. 

## Results

**Fig. 1:**
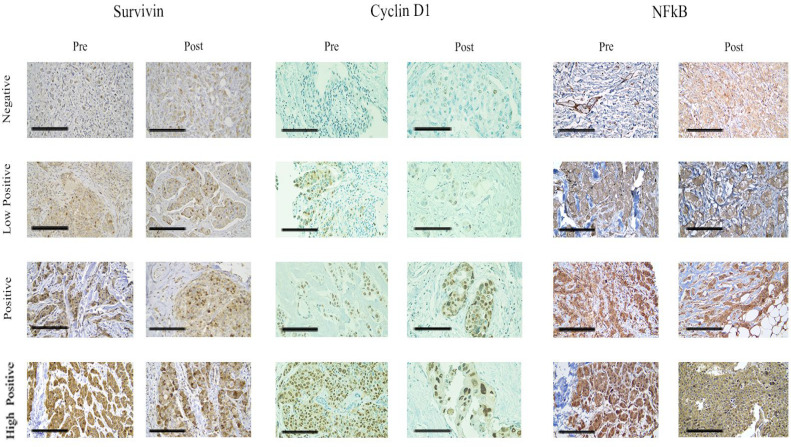
Immunohistochemistry staining for Survivin, Cyclin D1, and NFkB expression in the invasive breast cancer cells before and after the NC. All photos feature a 50 m scale marker. (400x magnification)

IHC was used to examine the Survivin, Cyclin D1, and NFkB expression levels in each of the 30 samples. Each tissue displayed the clinicopathologic characteristics listed in the Tables 1 and 2; both before and after the NC delivery. Figure 1 is the representative for the IHC staining outcomes. Different categories of the tumor cells, including those that stained negatively, weakly positively, positively, and highly positively, are represented in each picture. The brownness of the samples was measured with H-score. 

**Fig. 2:**
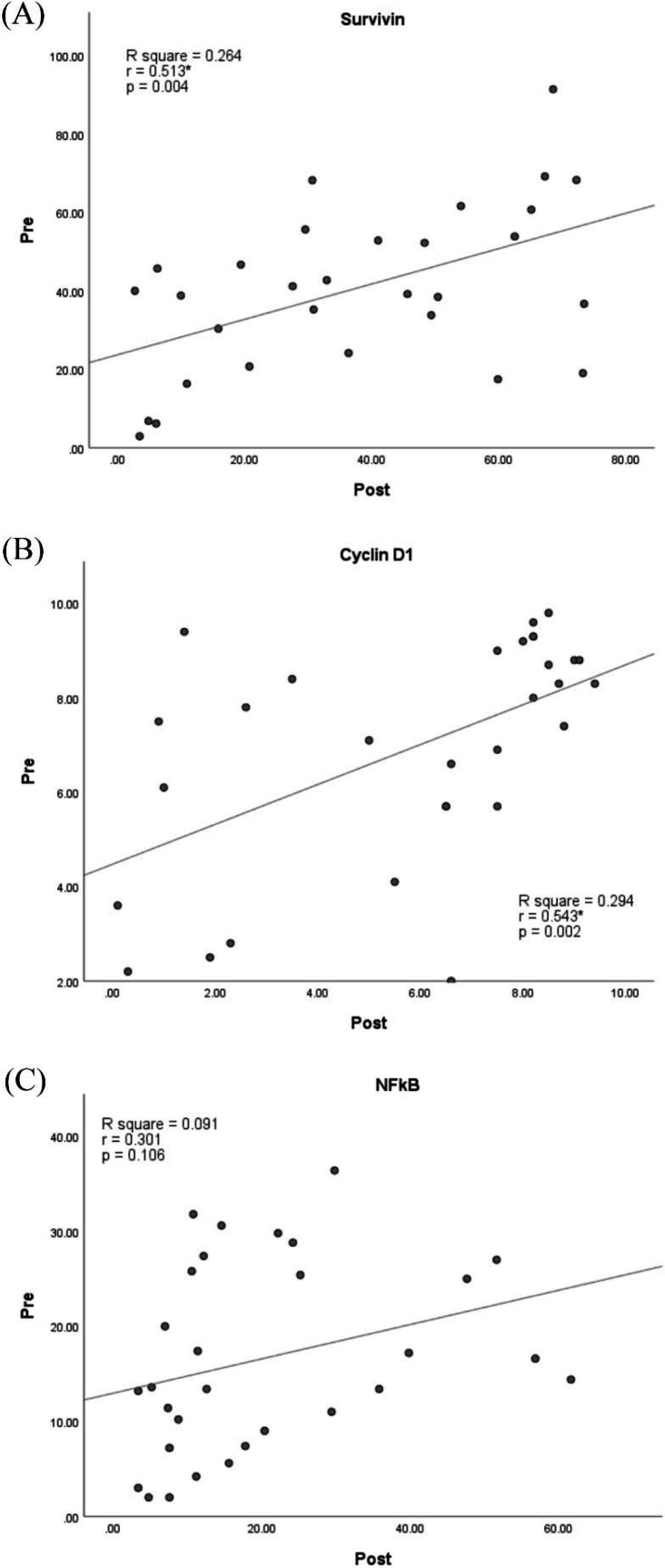
Correlation between (A) Survivin, (B) Cyclin D1, and (C) NFkB expression before and after the NC.

Tables 1 and 2 show the clinicopathological characteristics of each biomarker against the baseline parameters. No parameter showed a significant association with the changes in the expression of Survivin, Cyclin D1, and NFkB, both before and after the NC. On the other hand, there is a significant correlation between these biomarkers expression values before and after the NC. Figure 2A shows a significant moderate correlation (r=0.513, *P*=0.004) between before and after the NC Survivin expression. Simple linear regression analysis yielded the formula [Survivin Post] = 0.583 [Survivin Pre] + 13,617, with H-score as the Survivin expression value. Besides Survivin, Cyclin D1 also showed a significant moderate correlation (r=0.543, *P*=0.002) between before and after the NC (Figure 2B). Simple linear regression analysis resulted in the formula [Cyclin D1 Post]= 0.697 [Cyclin D1 Pre] + 1.022, with H-score as the Cyclin D1 expression value. However, NFkB expression was not observed to be correlated before and after the NC (Figure 2C).

**Table 1:** Clinicopathological characteristic before neoadjuvant chemotherapy administration

Variables	Category	Survivin Expression		*P*	Cyclin D1 Expression		*P*	NFkB Expression		*P*
		High (%)	Low (%)		High (%)	Low (%)		High (%)	Low (%)	
Age	<y.o.	6 (42.90%)	8 (57.10%)	0.696	3 (21.40%)	11 (78.60%)	0.513	8 (57.10%)	6 (42.90%)	0.510
	50 y.o.	8 (50.00%)	8 (50.00%)		2 (12.50%)	14 (87.50%)		11 (68.80%)	5 (31.30%)	
Tumor grade	1	2 (66.70%)	1 (33.30%)	0.547	0 (0.00%)	3 (100.00%)	0.549	2 (66.70%)	1 (33.30%)	0.524
	2	9 (50.00%)	9 (50.00%)		4 (22.20%)	14 (77.80%)		10 (55.60%)	8 (44.40%)	
	3	3 (33.30%)	6 (66.70%)		1 (11.10%)	8 (88.90%)		7 (77.80%)	2 (22.20%)	
ER status	Negative	8 (50.00%)	8 (50.00%)	0.696	4 (25.00%)	12 (75.00%)	0.190	10 (62.50%)	6 (37.50%)	0.919
	Positive	6 (42.90%)	8 (57.10%)		1 (7.10%)	13 (92.90%)		9 (64.30%)	5 (35.70%)	
PR status	Negative	10 (62.50%)	6 (37.50%)	0.282	3 (18.80%)	13 (81.30%)	0.743	11 (68.80%)	5 (31.30%)	0.510
	Positive	9 (64.30%)	5 (35.70%)		2 (14.30%)	12 (85.70%)		8 (57.10%)	6 (42.90%)	
HER2 status	Negative	8 (47.10%)	9 (52.90%)	0.961	2 (11.80%)	15 (88.20%)	0.410	11 (64.70%)	6 (35.30%)	0.858
	Positive	6 (46.20%)	7 (53.80%)		3 (23.10%)	10 (76.90%)		8 (61.50%)	5 (38.50%)	
Ki67 status	Negative	13 (50.00%)	13 (50.00%)	0.351	4 (15.40%)	22 (84.60%)	0.631	16 (61.50%)	10 (38.50%)	0.603
	Positive	1 (25.00%)	3 (75.00%)		1 (25.00%)	3 (75.00%)		3 (75.00%)	1 (25.00%)	

**ER,** estrogen receptor; **PR,** progesterone receptor, **HER2**, human epidermal growth factor receptor 2

Univariate analysis was performed using the chi-square test with continuity correlation. * P-value less than 0.05 is considered statistically significant

**Table 2:** Clinicopathological characteristic after neoadjuvant chemotherapy administration

Variables	Category	Survivin Expression		*P*	Cyclin D1 Expression		*P*	NFkB Expression		*P*
		High (%)	Low (%)		High (%)	Low (%)		High (%)	Low (%)	
Age	<y.o.	7 (50.00%)	7 (50.00%)	0.732	3 (21.40%)	11 (78.60%)	0.513	8 (57.10%)	6 (42.90%)	0.510
	50 y.o.	9 (56.30%)	7 (43.80%)		2 (12.50%)	14 (87.50%)		11 (68.80%)	5 (31.30%)	
Tumor grade	1	2 (50.00%)	2 (50.00%)	0.263	1 (25.00%)	3 (75.00%)	0.679	3 (75.00%)	1 (25.00%)	0.858
	2	6 (40.00%)	9 (60.00%)		3 (20.00%)	12 (80.00%)		9 (60.00%)	6 (40.00%)	
	3	8 (72.70%)	3 (27.30%)		1 (9.10%)	10 (90.90%)		7 (63.60%)	4 (36.40%)	
ER status	Negative	10 (62.50%)	6 (37.50%)	0.282	4 (25.00%)	12 (75.00%)	0.190	9 (56.30%)	7 (43.80%)	0.389
	Positive	6 (42.90%)	8 (57.10%)		1 (7.10%)	13 (92.90%)		10 (71.40%)	4 (28.60%)	
PR status	Negative	8 (50.00%)	8 (50.00%)	0.696	3 (18.80%)	13 (81.30%)	0.743	11 (68.80%)	5 (31.30%)	0.510
	Positive	8 (57.10%)	6 (42.90%)		2 (14.30%)	12 (85.70%)		8 (57.10%)	6 (42.90%)	
HER2 status	Negative	9 (52.90%)	8 (47.10%)	0.961	2 (11.80%)	15 (88.20%)	0.410	9 (52.90%)	8 (47.10%)	0.177
	Positive	7 (53.80%)	6 (46.20%)		3 (23.10%)	10 (76.90%)		10 (76.90%)	3 (23.10%)	
Ki67 status	Negative	14 (53.80%)	12 (46.20%)	0.886	4 (15.40%)	22 (84.60%)	0.631	15 (57.70%)	11 (42.30%)	0.102
	Positive	2 (50.00%)	2 (50.00%)		1 (25.00%)	3 (75.00%)		4 (100.00%)	0 (0.00%)	
ALNM	No	9 (60.00%)	6 (40.00%)	0.464	2 (13.30%)	13 (86.70%)	0.624	10 (66.70%)	5 (33.30%)	0.705
	Yes	7 (46.70%)	8 (53.30%)		3 (20.00%)	12 (80.00%)		9 (60.00%)	6 (40.00%)	
LVI	No	6 (42.90%)	8 (57.10%)	0.282	1 (7.10%)	13 (92.90%)	0.190	9 (64.30%)	5 (35.70%)	0.919
	Yes	10 (62.50%)	6 (37.50%)		4 (25.00%)	12 (75.00%)		10 (62.50%)	6 (37.50%)	
NC Response	Partial	15 (53.60%)	13 (46.40%)	0.992	5 (17.90%)	23 (82.10%)	0.513	18 (64.30%)	10 (35.70%)	0.685
	Complete	1 (50.00%)	1 (50.00%)		0 (0.00%)	2 (100.00%)		1 (50.00%)	1 (50.00%)	

**ALNM: **Axillary lymph node metastasis; **ER,** estrogen receptor;** HER2**, human epidermal growth factor receptor 2; **LVI**: Lymphovascular invasion; **NC:** neoadjuvant chemotherapy; **PR,** progesterone receptor

Univariate analysis was performed using the chi-square test with continuity correlation. * p-value less than 0.05 is considered statistically significant

## Discussion

The correlation between the expression values of Survivin, Cyclin D1, and NFkB before and after the NC obtained in this study aimed to answer the knowledge gap related to the potential use of these biomarkers as predictive IBC biomarkers. The correlation between before and after the NC expression indicates a significant change in the biomarker values. These changes could be attributed to the molecular processes underlying the action of NC on the individual biomarkers, both before and after administration. Before and after the NC, the link between Survivin and Cyclin D1 was determined to be statistically significant. However, similar findings were not obtained regarding NFkB. This indicates that, despite being on the same pathway, NC has less effect on the NFkB expression. This finding also implies that other factors may influence the action of NC on NFkB that do not affect changes in Survivin and Cyclin D1.

Survivin expression changes before and after the NC showed a significant correlation. These findings can be analyzed from the factors influencing this biomarker pathway. To sharpen the analysis, several confounding factors have been identified and analyzed in Tables 2 and 3. Factors that include patient demographic data, such as age and gender, IBC clinical data, such as tumor grade and NC response, histopathological data, such as lymph node metastasis and lymphovascular invasion, as well as immunohistochemistry data, such as staining of several other markers (ER, PR, HER2, and Ki67) were also examined. In addition to avoiding confounding analyses, these data were included as descriptive specifications of the IBC patients receiving NC administration at our center (22). After analysis, no clinicopathological characteristic parameterwas found to be associated with high and low Survivin expression, both before and after the NC. This indicates that before and after the NC, Survivin expression correlation was highly focused on only the effect of NC administration. With the confounding factors controlled, the correlation of Survivin expression changes could be analyzed in a focused manner. Furthermore, positive correlation was found in the Survivin expression changes. This shows an increase in the expression value of Survivin before and after the NC. This increase may be related to the administration of NC. This finding is fascinating because NC, which acts as IBC therapy, should inhibit and decrease Survivin expression, as an anti-apoptotic biomarker. An increase in the post-NC Survivin was also reported in the study of Rauch et al., (23). This chemotherapy-induced increase of Survivin may occur due to the ambivalent effect of the transcription factor p53 and NFkB (23, 24). Although p53 inhibits Survivin expression in resting cells, it turns out that NC, which induces DNA damage, actually activates Survivin (24). This mechanism is interesting because it seems that NC causes activation of the cell survival pathway under the influence of the NFkB gene. The simultaneous stimulation of p53 and NFkB in many cancers is compatible with the observed rise of Survivin after treatment (25, 26). Research by Rauch et al., (23) also showed an increase in Survivin in colorectal cancer treated with irinotecan. This observation was attributed to the increased stability of Survivin due to an arrest in the late S and G2/M phases (23). CDC2/CDK1 activation of Survivin at threonine 34 (T34) increases the protein stability (27). In addition, the overexpression of Survivin was also associated with resistance to NC (28, 29). This also explains why 93.33% of the patients in this study had a partial response to NC. 

In addition to Survivin, Cyclin D1 expression showed significant changes before and after the NC. Tables 2 and 3 display the results of comprehensive influencing factor analysis performed to specifically examine the variations in Cyclin D1 expression, as was done previously with Survivin. The results were also the same as Survivin, i.e., no clinicopathological characteristic parameter showed association with Cyclin D1 expression levels. Furthermore, a significant positive correlation between before and after the NC Cyclin D1 indicated increased Cyclin D1 expression after the NC administration. As a signaling protein in the NFkB pathway, the increase in Cyclin D1 can be explained by the exact similar mechanism to Survivin. This chemotherapy-induced increase of Cyclin D1 may occur due to the ambivalent effect of transcription factor p53 and NFkB (23, 24). Studies examining the correlation between Cyclin D1 expression level and chemotherapeutic response have shown contradictory findings (30). Some found an inverse correlation between Cyclin D1 expression and chemotherapy response, whereas others found the reverse (30). The proposed process incorporates the distinction between cancer cell types, the influence of chromosomal instability in a few malignancies, the stimulation for the excessive production of DNA repair proteins, and the response to the degree of DNA damage (30). Research by Irawan et al., supports the findings of this study, in which a high expression of Cyclin D1 in the NC-responsive nasopharyngeal carcinoma patients was reported (31). However, the opposite result was outlined by Feng et al., in the patients with head and neck squamous cell carcinoma (32). On the other hand, Bradford et al., showed no relationship between Cyclin D1 expression and response to the chemotherapy in laryngeal cancer patients (33). It was suggested that differences in tumor type, chemotherapy medications used, and the degree of cell damage generated by the cytostatics led to the inconsistent findings when examining the effect of cyclin D1 on chemotherapy (30). Similar to Survivin, Cyclin D1 in chemoresistance can also explain the distribution of the NC response in the patients, who almost all showed partial response.

Apart from Survivin and Cyclin D1, NFkB expression, as a regulatory gene for these two proteins, did not show a significant correlation before and after the NC. This finding can be explained this way that NFkB is a central gene influenced by several mechanisms. Based on the literature, activation of NFkB after NC seems to play a major role in activating downstream target genes, such as Survivin and Cyclin D1 (34). However, at the same time, NC also inhibits NFkB to suppress cancer cell proliferation (35). The existence of this dual role may be the cause of the insignificant value of NFkB in this study. Despite the non-significant correlation of the NFkB, the NC responses in the samples still showed the presence of chemoresistance. This may imply that NFkB overexpression, which occurred after the NC administration, resulted in the activation of the chemoresistance mechanism via Survivin and Cyclin D1, but was accompanied by a decrease in NFkB by the NC mechanism. However, further research is still needed to answer this hypothesis.

This research had strength and limitation points. The strength point of this study was the extensive analysis of three potential predictive biomarkers. In this study, before and after the NC correlation analyses were carried out by the first controlling for confounding factors. However, a balanced distribution of the patients with NC responses was not achieved even with an adequate sample size. Therefore, further research with a case-control design may be needed to approach the grouping of samples based on their NC responses. In addition, further research with larger sample size is also needed.

## Conclusion

The significant positive correlation of Survivin and Cyclin D1 expression between before and after the NC indicates the strong potential of these proteins as predictive biomarkers. This increase in biomarkers suggests a chemoresistance mechanism underlying IBC progression, given NC. Therefore, further research is needed regarding the effect of these biomarkers on the specific chemotherapy response.

## Conflict of Interest

The authors declared no conflict of interests.
